# Engineering *Escherichia coli* to convert acetic acid to β-caryophyllene

**DOI:** 10.1186/s12934-016-0475-x

**Published:** 2016-05-05

**Authors:** Jianming Yang, Qingjuan Nie

**Affiliations:** Key Lab of Plant Biotechnology in Universities of Shandong Province; College of Life Sciences, Qingdao Agricultural University, Qingdao, 266109 China; Key Lab of Applied Mycology, College of Life Sciences, Qingdao Agricultural University, No.700 Changcheng Road, Chengyang District, Qingdao, 266109 China; Foreign Languages School, Qingdao Agricultural University, Qingdao, 266109 China

**Keywords:** β-caryophyllene, Acetic acid, MVA pathway, *E. coli*

## Abstract

**Background:**

Under aerobic conditions, acetic acid is the major byproduct produced by *E. coli* during the fermentation. And acetic acid is detrimental to cell growth as it destroys transmembrane pH gradients. Hence, how to reduce the production of acetic acid and how to utilize it as a feedstock are of intriguing interest. In this study, we provided an evidence to produce β-caryophyllene by the engineered *E. coli* using acetic acid as the only carbon source.

**Results:**

Firstly, to construct the robust acetate-utilizing strain, acetyl-CoA synthases from three different sources were introduced and screened in the *E. coli*. Secondly, to establish the engineered strains converting acetic acid to β-caryophyllene, acetyl-CoA synthase (ACS), β-caryophyllene synthase (QHS1) and geranyl diphosphate synthase (GPPS2) were co-expressed in the *E. coli* cells. Thirdly, to further enhance β-caryophyllene production from acetic acid, the heterologous MVA pathway was introduced into the cells. What’s more, acetoacetyl-CoA synthase (AACS) was also expressed in the cells to increase the precursor acetoacetyl-CoA and accordingly resulted in the increase of β-caryophyllene. The final genetically modified strain, YJM67, could accumulate the production of biomass and β-caryophyllene up to 12.6 and 1.05 g/L during 72 h, respectively, with a specific productivity of 1.15 mg h^−1^ g^−1^ dry cells, and the conversion efficiency of acetic acid to β-caryophyllene (gram to gram) reached 2.1 %. The yield of β-caryophyllene on acetic acid of this strain also reached approximately 5.6 % of the theoretical yield.

**Conclusions:**

In the present study, a novel biosynthetic pathway for β-caryophyllene has been investigated by means of conversion of acetic acid to β-caryophyllene using an engineered *Escherichia coli*. This was the first successful attempt in β-caryophyllene production by *E. coli* using acetic acid as the only carbon source. Therefore, we have provided a new metabolic engineering tool for β-caryophyllene synthesis.

## Background

β-caryophyllene, a common sesquiterpene that is widely found in plants [1, 2], is being considered for use as a component in the next generation aircraft fuel [3–5]. Conventionally, like other sesquiterpenes, extraction from plants and chemical synthesis have always been accepted as the common options for the production of β-caryophyllene on a large scale, yet, both methods have their own disadvantages. Low concentration and poor recovery yields [6, 7] make the isolation of β-caryophyllene from plants both infeasible and uneconomical. Meanwhile, complexity of the process, the diminishing reserve of petroleum and increasingly serious environmental problems are impelling us to move our eyes on the microbial production of β-caryophyllene, which can utilize renewable glucose derived from lignocellulose. In addition, compared with traditional methods, β-caryophyllene produced by microbial fermentation expects to be a good alternative mainly due to its environmental friendliness and sustainable development.

However, in spite of the above advantages in the microbial synthesis, there are also some bottlenecks limiting its application. For instance, under aerobic conditions, acetic acid (HAc) is the major byproduct produced during fermentation in high cell density cultures. Unfortunately, high concentration of HAc is detrimental to cell growth as it destroys transmembrane pH gradients. This negatively affects internal osmotic pressure, recombinant protein synthesis and biomass formation [8, 9]. In addition, the production of the byproduct HAc accordingly decreases the carbon effective utilization and the yield of target product.

In the previous studies, acetic acid had been considered to be used as a feedstock for production of biofuel or biochemical since it could be generated from a variety of cheap sources. (1) Methanol carbonylation is an effective method for HAc production [10]. (2) HAc is a major product during syngas fermentation [11, 12] and a byproduct from hydrolysis (under acid or alkali pretreatment [13] or pyrolysis of lignocellulosic biomass [14]. (3) Methane from natural gas or biogas can be converted to HAc [15]. (4) HAc is also an intermediate from anaerobic digestion of organic wastes. [16].*Cryptococcus curvatus* had been demonstrated to use HAc as a major carbon source for lipid production [17–19] and some *Clostridium* species could utilize HAc and sugar concurrently for alcohols or butyrate biosynthesis [20, 21]. Hence, strengthening the ability of *E. coli* to assimilate HAc would lessen harmful effects of HAc, recycle wasted carbon, and enhance carbon flux toward the desired pathways. However, there are no reports on metabolic engineering of the *E. coli* strain which can utilize HAc as the main carbon source to produce β-caryophyllene.

**Fig. 1:**
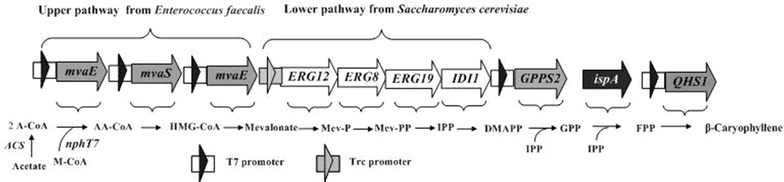
Production of β-caryophyllene via the MVA-mediated pathway used in this study. Gene symbols and the enzymes they encode (all genes marked with* black arrows* were from *Enterococcus faecalis*, all genes marked with* white arrows* were isolated from *Saccharomyces cerevisiae*, the gene marked with* gray arrows* and* black characters* were derived from *Abies grandis* or *Artemisia annua*, and the gene marked with* gray arrows* and* white characters* were native genes in *E. coli*). Enzymes in MVA pathway: MvaE(acetyl-CoA acetyltransferase/HMG-CoA reductase) and MvaS(HMG-CoA synthase) from *Enterococcus faecalis*; ERG12(mevalonate kinase), ERG8(phosphomevalonate kinase), ERG19(mevalonate pyrophosphate decarboxylase) and IDI1(IPP isomerase) from *Saccharomyces cerevisiae*; GPPS2(geranyl diphosphate synthase from *Abies grandis*); QHS1(β-caryophyllene synthase from *Artemisia annua*); IspA(GPP synthase/FPP synthase) from *E. coli*. ACS, nphT7. Intermediates in MVA pathway: A-CoA, acetyl-CoA; AA-CoA, acetoacetyl-CoA; HMG-CoA, hydroxymethylglutaryl-CoA; Mev-P, mevalonate 5-phosphate; Mev-PP, mevalonate pyrophosphate. *IPP* isopentenyl pyrophosphate; *DMAPP* dimethylallyl pyrophosphate; *GPP* geranyl diphosphate; *FPP* farnesyl diphosphate

In the present study, we are making an attempt to use acetic acid (HAc) as a feedstock for production of β-caryophyllene. A multi-step metabolic engineering strategy (Fig. 1) was employed to enhance the ability to utilizing HAc, increase the supply of some precursors such as IPP, DMAPP, GPP and acetoacetyl-CoA, which ultimately led to the increase in the β-caryophyllene production. The final genetically modified strain, YJM67, cultured under the fed-batch fermentation condition, could accumulate the yield of biomass and β-caryophyllene up to 12.6 and 1.05 g/L during 72 h, respectively, and the conversion efficiency of HAc to β-caryophyllene (gram to gram) reached 2.1 %, which was the first successful attempt in β-caryophyllene production by *E. coli* using the HAc as the only carbon source. Therefore, we have provided a new metabolic engineering tool for β-caryophyllene synthesis.

## Results and discussion

### Engineered the high efficiency pathway for HAc utilization in *E. coli*

HAc is employed as a potential substrate for production of biomass, biofuels and value added chemicals. However, *E. coli* has a restricted ability to use HAc for biomass growth. The metabolic pathways which *E. coli* utilizes HAc are mainly via AMP-forming acetyl-CoA synthetase (the acs pathway) and phosphotransacetylase/acetate kinase (the reversible PTA-ACKA pathway) [9, 22]. In the previous study, it has proven that overexpressing the single *acs* gene along with maintaining the native HAc pathways was the best strategy for HAc assimilation in *E. coli* [23, 24]. A suitable method to optimize pathway efficiency may be to use genes from different organisms [25]. In this paper, the acetyl-CoA synthase enzymes from native *E. coli*, *Salmonella typhimurium* LT2 and *Acetobacter pasteurianus* were assessed to utilize HAc for cell growth.

**Fig. 2:**
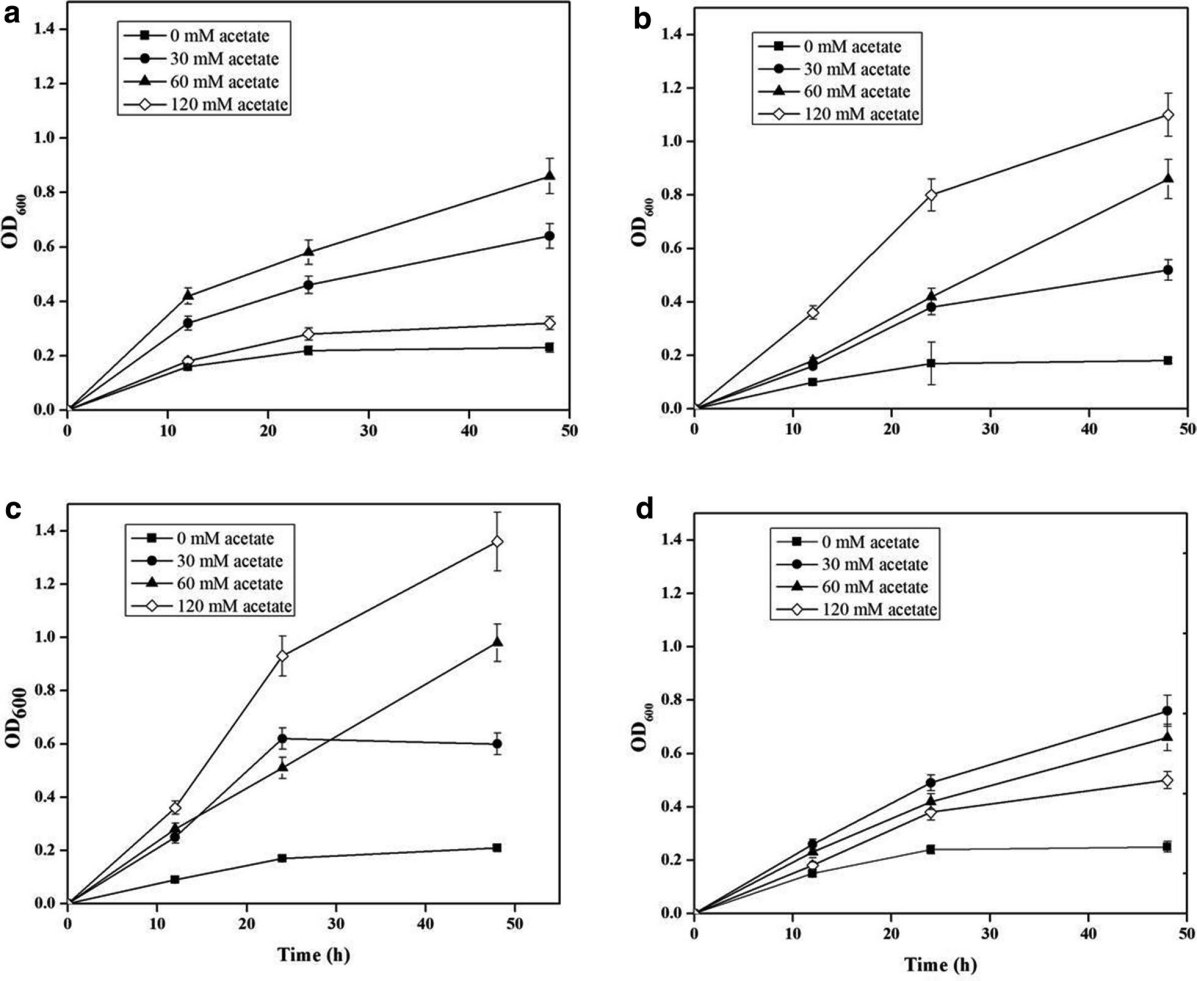
Comparative growths of the engineered strains using acetic acid. The engineered strains *E. coli* BL21(DE3)/pCOLADUet-1 (**a**), BL21(DE3)/pYJM60 (**b**), BL21(DE3)/pYJM62 (**c**) and BL21(DE3)/pYJM61 (**d**) grew on different concentrations of acetate. The OD_600_ was measured spectrophotometrically. The experiment was performed in triplicate

Therefore, in this paper, to construct the acetate utilization pathway in *E. coli*, the different *ACS* genes were screened. The gene *ACS*_*EC*_ from *E. coli*, *ACS*_*ST*_ from *S. typhimurium* LT2 and *ACS*_*AP*_ from *A. pasteurianus* were cloned into the plasmid pCOLDuet-1 forming the plasmids pYJM60, pYJM61 and pYJM62, respectively. The different strains containing pYJM60, pYJM61 and pYJM62 named as YJM60, YJM61 and YJM62, were cultured using M9 medium with different concentrations of sodium acetate. As shown in Fig. 2, the results showed that the strain containing *ACS*_*AP*_ gene grow best among three strains, also suggested that the enzyme from *A. pasteurianus* was the most efficient in the conversion of acetate into acetyl-CoA.

### Biosynthesis of β-caryophyllene from HAc using native MEP pathway

Farnesyl diphosphate (FPP), generated from either the DXP or MVA pathway [26, 27], could be catalyzed by the β-caryophyllene synthase into β-caryophyllene [28, 29]. Nevertheless, *E. coli* harbors MEP pathway, and is not capable of producing β-caryophyllene on its own due to the lack of β-caryophyllene synthase. In the study, to produce β-caryophyllene, the optimized *QHS1* gene from *Artemisia annua* was overexpressed along with ACS_AP_ enzyme from *A. pasteurianus* in the engineered *E. coli* (YJM63 containing pACY-*QHS1*/pCOL-*ACS*_*AP*_). Firstly, the recombinant strain YJM63 was grown in the M9 medium with acetate as the carbon source, and the target product β-caryophyllene was not detected. While the engineered strain cultured in the M9 medium with 2 g/L glucose as the carbon source, it could produce 56 ± 6 μg/L β-caryophyllene. No β-caryophyllene production was detected in the control strain harboring the empty vector pACYCDUet-1/pCOL-*ACS*_*AP*_. Based on the results, using the native MEP pathway, *QHS1* gene from *A. annua* and *ACS*_*AP*_ gene from *A. pasteurianus*, a novel biosynthetic pathway for the β-caryophyllene production with acetate as the carbon source has been successfully established in the *E. coli*. However, the effectiveness of the whole metabolic pathway for β-caryophyllene production is too low. The main reason might be short of other important precursors such as DMAPP, IPP and GPP.

Geranyl diphosphate (GPP), the key precursor of sesquiterpene production, is catalyzed from the condensation of dimethylallyl diphosphate and isopentenyl diphosphate by GPP synthase [30]. In this paper, to enhance the supply of GPP and accordingly to increase β-caryophyllene production, the GPP synthase of *Abies grandis* was co-expressed in the cell along with *QHS1* and *ACS*_*AP*_ genes and formed into the engineered strain YJM64 (*E. coli* BL21(DE3) containing pACY-*QHS1*-*GPPS2*/pCOL-*ACS*_*AP*_). According to the GC analysis results, after 24 h of culture, 102 ± 9 μg/L β-caryophyllene was produced by the *E. coli* strain YJM64. The results demonstrated that the heterologous expression of GPP synthase (GPPS) is beneficial to the β-caryophyllene production. This finding is corresponding to previous studies on the production of other terpenes such as α-pinene and sabinene [31, 32].

### Establishing a MVA-mediated biosynthetic pathway for β-caryophyllene production from acetate

Despite of progress made in the β-caryophyllene biosynthesis using the native MEP pathway, the yield of β-caryophyllene was so low that it is not economic and feasible for industrial application. In previous studies, the hybrid MVA pathway has proven to be efficient on the biosynthesis of DMAPP and IPP [33, 34] which are the precursors of all terpenes. Based on these findings, a hypothesis can be put forward that the MVA pathway might be more efficient than the MEP pathway in β-caryophyllene production using acetate as carbon source.

In an attempt to increase the production of β-caryophyllene, the hybrid MVA pathway was introduced into *E. coli* and overexpressed along with *QHS1* from *A. annua*, *GPPS2* gene from *A. grandis* and *ACS*_*AP*_ gene from *A. pasteurianus*. As expected, the recombinant strain YJM66 (pACY-*mvaE*-*mvaS*-*QHS1*-*GPPS2*/pTrc-Low/pCOL-*ACS*_*AP*_) carrying hybrid MVA pathway could accumulate 8 ± 0.75 mg/L β-caryophyllene, which is about eight-fold higher than that (102 ± 9 μg/L) produced by the control strain YJM64 (pACY-*QHS1*-*GPPS2*/pCOL-*ACS*_*AP*_) without the hybrid MVA pathway. According to the data obtained, it may be easier to make a conclusion that the hybrid MVA pathway is conducive to the β-caryophyllene biosynthesis.

### The effect of AACS on β-caryophyllene production from Acetate

In recent studies, acetoacetyl-CoA synthase (AACS) from *Streptomyces sp. strain CL190* has been proven to catalyze a single condensation of acetyl-CoA and malonyl-CoA to form acetoacetyl-CoA [35]. Unlike acetoacetyl-CoA thiolase (EC 2.3.1.9), which also synthesize acetoacetyl-CoA by reversible nondecarboxylative condensation of two molecules of acetyl-CoA and prefers acetoacetyl-CoA thiolysis to acetoacetyl-CoA synthesis, NphT7 proves no thiolysis activity against acetoacetyl-CoA, and since NphT7-catalyzed acetoacetyl-CoA synthesis is essentially an energy-favored reaction, NphT7 could be an ideal enzyme to supply acetoacetyl-CoA in cells [36].

When the AACS-encoding gene (nphT7) was expressed combining with the HMG-CoA synthase gene and the HMG-CoA reductase gene in *E.coli*, the engineered strain could achieve 3.5-fold higher production of mevalonate than the control strain without nphT7 expression [36]. Based on these findings, it may be hypothesized that overexpression of acetoacetyl-CoA synthase (AACS) in β-caryophyllene-producing strain would enhance β-caryophyllene productivity.

In the paper, to further increase β-caryophyllene production, the AACS-encoding gene (nphT7) was overexpressed in the β-caryophyllene producing *E. coli* YJM67 (pACY-*mvaE*-*mvaS*-*QHS1*-*GPPS2*/pTrc-Low/pCOL-*ACS*_*AP*_-*nphT7*). After cultured in the M9 medium with 60 mM acetate as the carbon source for 24 h, the yield of β-caryophyllene reached 22 ± 1.8 mg/L, which is 2.75-fold to the control strain YJM66 without *nphT7* gene expression (8 ± 0.75 mg/L). The results demonstrated that acetoacetyl-CoA synthase is helpful to increase acetoacetyl-CoA in cells and accordingly to enhance β-caryophyllene production.

### Effect of the type and concentration of the nitrogen source on β-caryophyllene production

**Fig. 3:**
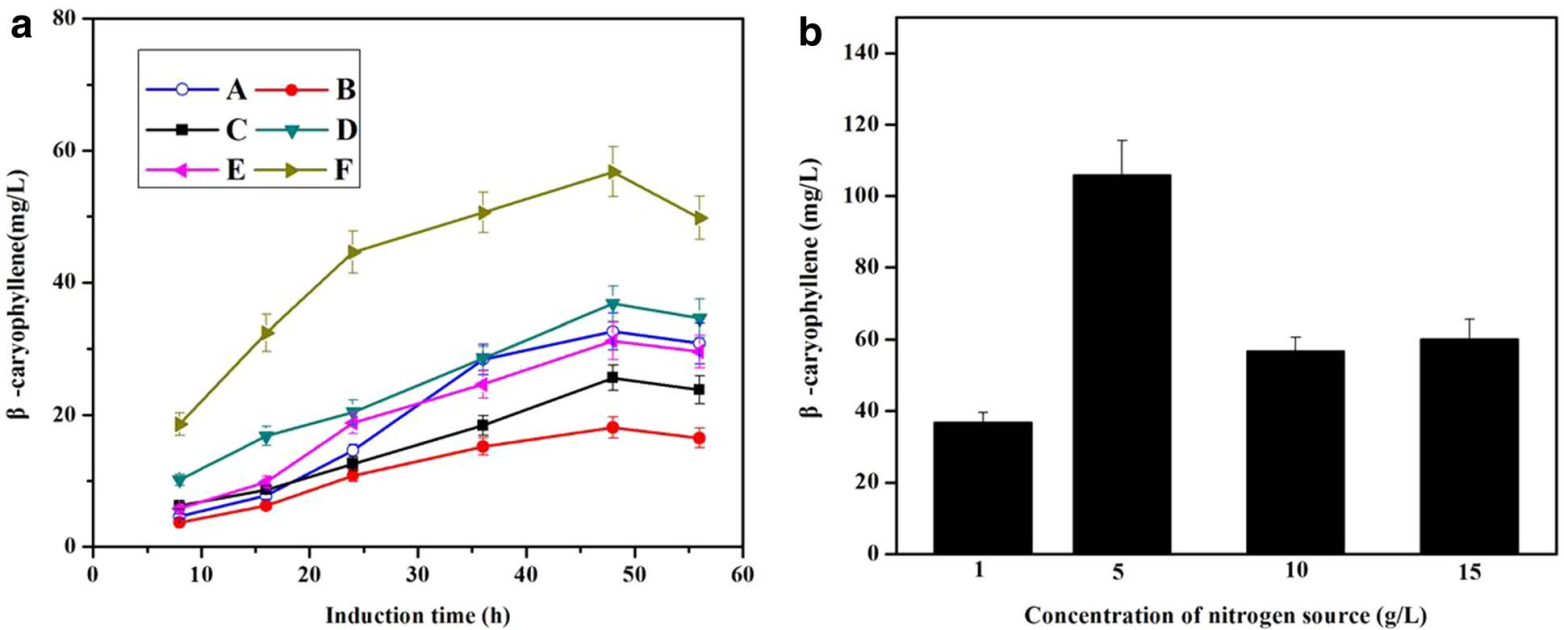
The effects of types and concentrations of nitrogen source on β-caryophyllene production by YJM67. **a** The Effect of different organic nitrogen source on β-caryophyllene production by YJM67. **a** Beef extract (Aladdin, ○); **b** yeast extract powder (Beijing AoBoXing Bio-Tech Co., Ltd, ●); **c** beef extract powder (MDBio, Inc, ■); **d** beef extract (Beijing Shuangxuan Microbe Culture Medium Products Factory, ▼); **e** beef extract (Sinopharm Chemical Reagent Co., Ltd, ◄); **f** beef extract (solarbio, ►). **a** The Effect of concentration of nitrogen source on β-caryophyllene production by YJM67. When OD_600_ reached 0.6–0.9, cultures were induced for 56 h using IPTG in shake-flasks. All the experiments were carried out in triplicates. Optimized conditions: Nitrogen sources, beef power; concentrations of nitrogen source, 5 g/L

The source of the nitrogen in the medium plays an important role in improving the biosynthesis of desired product [37, 38]. To investigate the effect of type and concentration of organic nitrogen source on β-caryophyllene production, six different organic nitrogen sources were chosen and valuated (Fig. 3a). Among the organic nitrogen supplements tried, the Solarbio beef extract allowed a significantly higher β-caryophyllene production than the other organic nitrogen sources.

Then to determine the optimum concentration of Solarbio beef extract, various beef extract concentrations, ranging from 1 to 15 g/L, were tested. According to the data shown in Fig. 3b, the maximum production of β-caryophyllene reached 106 ± 9.6 mg/L, which was approximately five times as much as the production before optimization (22 ± 1.8 mg/L).

Based on the above data, the most suitable types and concentration of nitrogen source for β-caryophyllene production using the engineered strain YJM67 were 5 g/L Solarbio beef extract.

### Lab-scale batch production of β-caryophyllene

**Fig. 4:**
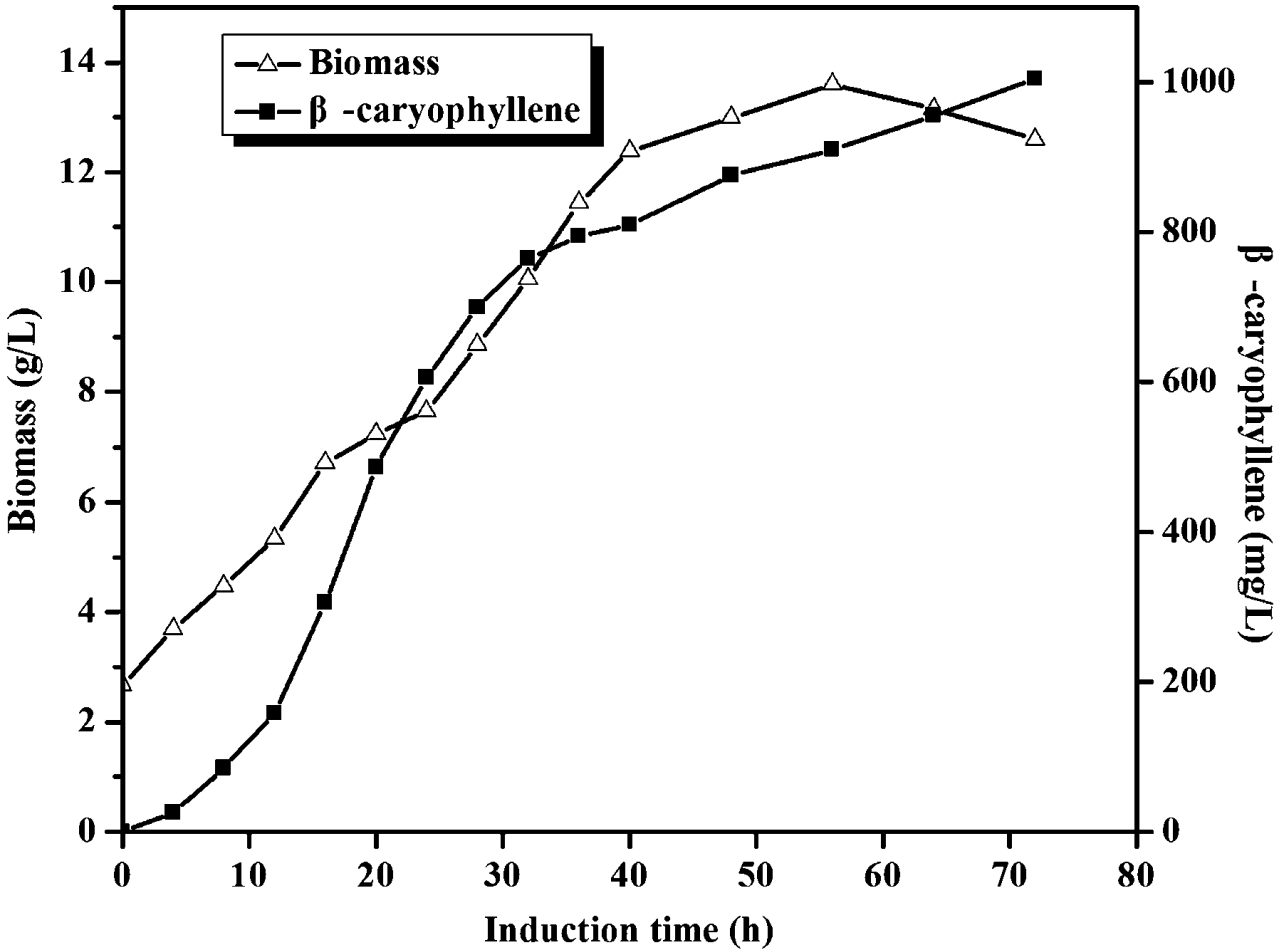
The time course of β-caryophyllene production by YJM67. Biomass (Δ) and β-caryophyllene accumulation (■) in YJM67. Induction was carried out when OD_600_ reached about 6 at 30 °C. Other experimental conditions are described in section “Fed-Batch Fermentation”

To scale up β-caryophyllene production from HAc, a pH-coupled HAc fed batch fermentation, which can gradually add HAc to the fermentation medium, was performed in a 5-L-scale laboratory batch reactor, in which was grown our most optimized strain, YJM67. Based on the flask condition, the final engineered strain YJM67 was cultured in the 5-L-scale laboratory batch reactor with the minimal medium plus 5 g/L beaf extract. As seen in the Fig. 4, the production of biomass and β-caryophyllene increased substantially without obvious lag phase and their maximum concentrations reached 12.6 and 1.05 g/L at 72 h, respectively, with a specific productivity of 1.15 mg h^−1^ g^−1^ dry cells, and the conversion efficiency of HAc to β-caryophyllene (gram to gram) reached 2.1 %. The yield of β-caryophyllene on HAc of this strain also reached approximately 5.6 % of the theoretical yield (of 37.81 %) based on the following formula: 9 HAc → 9 Acetyl-CoA → β-caryophyllene.

These results suggest great potential for this engineered *E*. *coli* strain in the production of β-caryophyllene on a large scale.

## Conclusions

In the paper, a novel biosynthetic pathway for β-caryophyllene has been constructed by assembling acetyl-CoA synthase, acetoacetyl-CoA synthase, β-caryophyllene synthase and the hybrid MVA pathway in the engineered *E. coli* strain. The final genetically modified strain, YJM67, could accumulate the production of biomass and β-caryophyllene up to 12.6 and 1.05 g/L during 72 h, respectively, and the conversion efficiency of HAc to β-caryophyllene (gram to gram) reached 2.1 %. To our knowledge, this was the first successful attempt in β-caryophyllene production by *E. coli* using the HAc as the only carbon source. Therefore, we have opened up a way to produce β-caryophyllene or other biochemicals and bioenergy on the HAc replacing glucose as a carbon source.

## Methods

### Strains, plasmids and culture conditions

All strains and plasmids used in this study are listed in Table 1. *E. coli* DH5a was employed in all the cloning work. *E. coli* BL21(DE3) was used for the product biosynthesis. For β-caryophyllene production, different strains were cultivated in shake-flask or fed-batch fermentation conditions with modified M9 minimal medium containing different concentrations of sodium acetate, beef extract 5 g/L. M9 minimal medium consists of Na_2_HPO_4_ 6, KH_2_PO_4_ 3, NH_4_Cl 1, NaCl 0.5, MgSO_4_ 0.24 g/L. If necessary, suitable antibiotics were added to the culture medium at the following concentrations: ampicillin (100 µg/ml), kanamycin (50 µg/ml), and chloramphenicol (34 µg/ml).

**Table 1:** Strains and plasmids used in this study

Name	Relevant characteristics	References
Strains		
*E.coli* BL21(DE3)	F^−^ *ompT hsd*S_B_ (r_B_^−^m_B_^−^) *gal dcm rne*131 λ(DE3)	Invitrogen
*E.coli* DH5α	*deoR*, recA1, endA1, hsdR17(rk-,mk +), phoA, supE44, λ-, thi-1, gyrA96, relA1	Takara
YJM60	*E.coli* BL21(DE3)/pYJM60	This study
YJM61	*E.coli* BL21(DE3)/pYJM61	This study
YJM62	*E.coli* BL21(DE3)/pYJM62	This study
YJM63	*E.coli* BL21(DE3)/pYJM63, pYJM62	This study
YJM64	*E.coli* BL21(DE3)/pYJM64, pYJM62	This study
YJM66	*E.coli* BL21(DE3)/pYJM66, pYJM14, pYJM62	This study
YJM67	*E.coli* BL21(DE3)/pYJM66, pYJM14, pYJM67	This study
Plasmids		
pACYCDuet-1	P15A (pACYC184), Cm^r^	Novagen
pTrcHis2B	pBR322 origin, Amp^r^	Invitrogen
pCOLADuet-1	ColA *ori*, *lacI* T7*lac*, Kan^r^	Novagen
pYJM14	pTrcHis2B carrying *ERG12*, *ERG8*, *ERG19* and *IDI1* from *Saccharomyces cerevisiae*	[29]
pYJM16	pACYCDuet-1 carrying *mvaE and mvaS* from *Enterococcus faecalis*	[28]
pYJM60	pCOLADuet-1 carrying *ACS*_*EC*_ from *E. coli*	This study
pYJM61	pCOLADuet-1 carrying *ACS*_*ST*_ from *Salmonella typhimurium* LT2	This study
pYJM62	pCOLADuet-1 carrying *ACS*_*AP*_ from *Acetobacter pasteurianus*	This study
pYJM63	pACYCDuet-1 carrying *QHS1* from *Artemisia annua*	This study
pYJM64	pACYCDuet-1 carrying *QHS1* from *Artemisia annua*, *GPPS2* from *Abies grandis*	This study
pYJM65	pACYCDuet-1 carrying *QHS1* from *Artemisia annua*, *mvaE and mvaS* from *Enterococcus faecalis*	This study
pYJM66	pACYCDuet-1 carrying *QHS1* from *Artemisia annua*, *mvaE and mvaS* from *Enterococcus faecalis*, *GPPS2* from *Abies grandis*	This study
pYJM67	pCOLADuet-1 carrying *ACS*_*AP*_ from *Acetobacter pasteurianus,* *nphT7* from *Streptomyces* sp. strain CL190	This study

### Plasmid construction

The nucleotide sequence of acetyl-CoA synthetase genes from *E. coli* (*ACS*_*EC*_, GenBank No. ACB05066.1) was obtained by PCR amplification using its genomic DNA as the template. The nucleotide sequences of acetyl-CoA synthetase genes from *Salmonella typhimurium* LT2 (*ACS*_*ST*_, GenBank No. AAL23099.1) and from *Acetobacter pasteurianus* (*ACS*_*AP*_, GenBank No. AKR48484.1) were chemically synthesized by Genray Company with plasmid pGH as the vector (called pGH-*ACS*_*ST*_*and* called pGH-*ACS*_*AP*_).

The nucleotide sequence of acetoacetyl CoA synthase from *Streptomyces* sp. CL190 (*nphT7*, GenBank No. AB540131.1) was chemically synthesized by Genray Company with plasmid pGH as the vector (called pGH- *nphT7*).

The nucleotide sequences of β-caryophyllene synthase genes from *Artemisia annua* (*QHS1*, GenBank No. AF472361.1) was analyzed using online software (http://www.genscript.com/cgi-bin/tools/rare_codon_analysis) and optimized to the preferred codon usage of *E. coli* (http://www.jcat.de/). The codon-optimized *QHS1* gene was chemically synthesized by Genray Company with plasmid pGH as the vector (called pGH-*QHS1*).

The geranyl diphosphate synthase (*GPPS2*) gene (GenBank No. AF513112) from *Abies grandis* was analyzed by online software (http://www.genscript.com/cgi-bin/tools/rare_codon_analysis) and optimized to the preferred codon usage of *E. coli* (http://www.jcat.de/). The codon-optimized *GPPS2* gene was synthesized by Genray Company with the plasmid pGH as the vector (named pGH-*GPPS2*).

To get the plasmid pYJM60 (pCOL-*ACS*_*EC*_), pYJM61 (pCOL-*ACS*_*ST*_), pYJM62 (pCOL-*ACS*_*AP*_), the *ACS*_*EC*_, *ACS*_*ST*_ and *ACS*_*AP*_ genes were ligated into pCOLADuet-1 using NcoI and BamHI, NcoI and BamHI, BamHI and EcoRI, respectively.

The *QHS1* gene fragment was obtained by digestion of pGH-*QHS1* with BglII and FseI and then ligated into the corresponding sites of pACYCDuet-1 to create pYJM63 (pACY-*QHS1*).

**Fig. 5:**
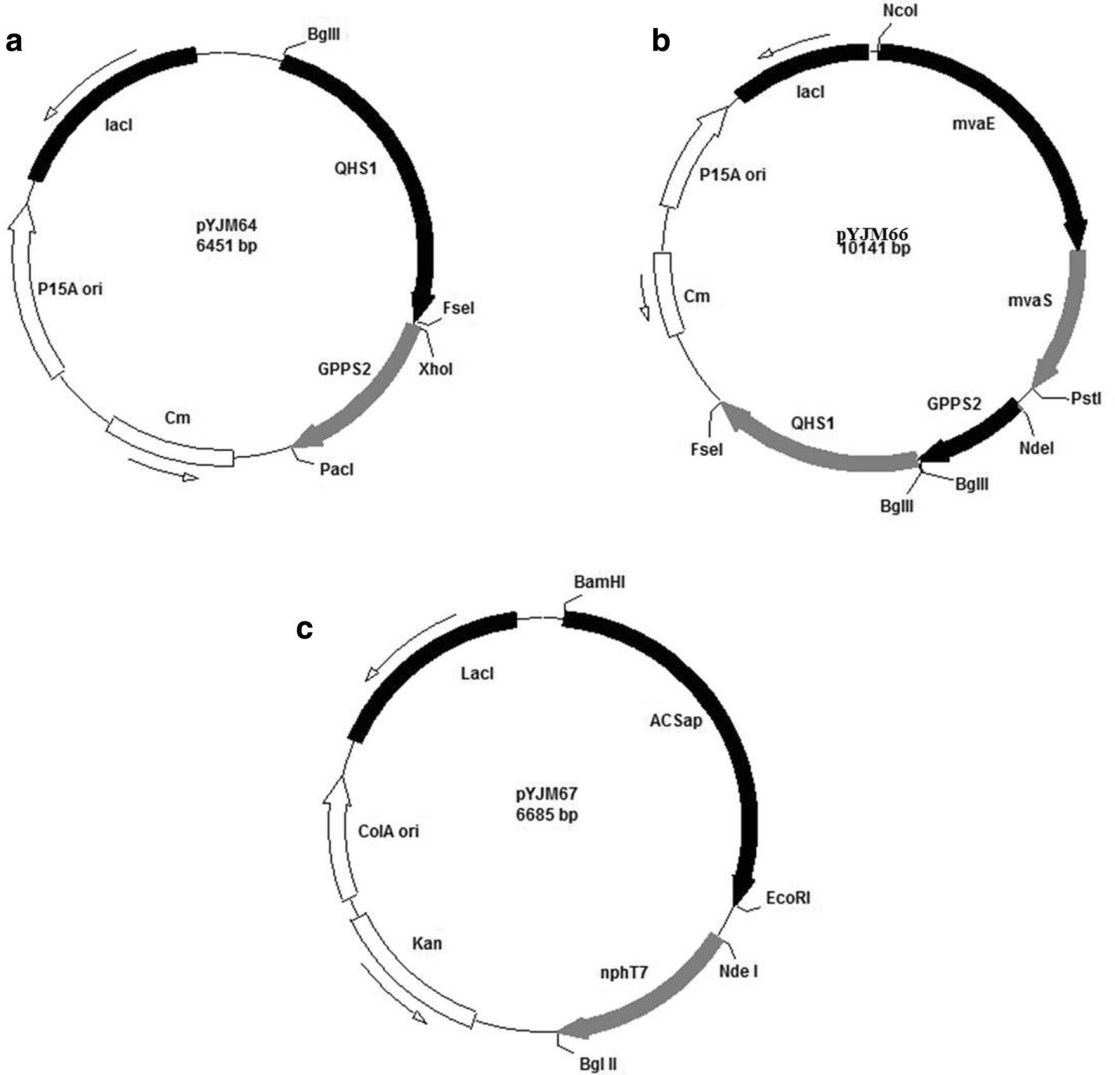
Plasmids used in this study. **a** Represented the plasmid pYJM64 harboring *QHS1* from *Artemisia annua*, *GPPS2* from *Abies grandis*; **b** represented the plasmid pYJM66 carrying *QHS1* from *Artemisia annua*, *mvaE and mvaS* from *Enterococcus faecalis*, *GPPS2* from *Abies grandis*; **c** represented the plasmid pYJM67 containing *ACS*_*AP*_ from *Acetobacter pasteurianus,* *nphT7* from *Streptomyces* sp. strain CL190

To get the plasmid pYJM64, The *GPPS2* gene fragment was obtained by digestion of pGH-GPPS2 with XhoI and PacI, and then ligated into the corresponding sites of pYJM63 to create pYJM64 (pACY-*QHS1*-*GPPS2*) (Fig. 5a).

To get the plasmid pYJM65, the *QHS1* gene fragment was obtained by excision from pYJM63 with BglII and FseI and was introduced into the corresponding sites of pYJM16 to create pYJM65 (pACY-*mvaE*-*mvaS*-*QHS1*).

The *GPPS2* gene fragment was obtained by digestion of pGH-GPPS2 with XhoI and PacI, and then ligated into the corresponding sites of pYJM65 to create pYJM66 (pACY-*mvaE*-*mvaS*-*QHS1*-*GPPS2*) (Fig. 5b).

To get the plasmid pYJM67, the *nphT7* gene fragment was obtained by digestion of pGH-*nphT7* using Nde I and Bgl II enzymes, and then ligated into the pYJM67(pCOL-*ACS*_*AP*_-*nphT7*) (Fig. 5c).

The plasmid pYJM16 was created based on pACYCDuet-1 by introducing *mvaE* and *mvaS* from *Enterococcus faecalis* [39]. The plasmid pYJM14 was constructed on pTrcHis2B by introducing the *ERG8*, *ERG12*, *ERG19* and *IDI1* from *S. Cerevisiae* [40].

### β-caryophyllene production under flask conditions

The engineered *E. coli* strains was cultivated in 50 ml fermentation medium as described above plus chloramphenicol (34 µg/ml) or/and ampicillin (100 µg/ml) or/and kanamycin (50 µg/ml) with shaking at 180 rpm. To induce gene expression at 30 °C for 56 h, 0.25 mM IPTG was added into the medium when the OD_600_ achieved about 0.6. a 1 ml headspace gas sample of β-caryophyllene was extracted from the culture broth based on the procedure of Reinsvold et al. [41] and measured by GC-FID. Different concentrations of β-caryophyllene produced by the strains were calculated by means of converting the GC peak area to μg or mg of β-caryophyllene via a calibration curve.

GC analysis was performed on an Agilent 7890A equipped with a flame ionization detector (FID) and an HP-INNOWAX column (25 m × 250 μm × 0.2 μm). N2 was used as carrier gas with a linear velocity of 1 ml/min. The column temperature profile was 50 °C for 0.5 min, 4 °C/min increase to 70 °C, 15 °C/min increase to 150 °C, 25 °C/min increase to 250 °C and 250 °C for 5 min. The product was characterized by direct comparison with an authentic standard (Sigma-Aldrich). The peak area was converted into β-caryophyllene concentration according to a standard curve plotted with a set of known concentrations of β-caryophyllene. All the error bars presented in the results indicated the standard deviation from the mean (n = 3).

### Optimization of type and concentration of organic nitrogen source

#### Effect of type of organic nitrogen source

The shake-flask cultures were incubated in M9 minimum medium with different organic nitrogen sources (10 g/L) (beef extract (solarbio), beef extract (Aladdin), beef extract (Beijing Shuangxuan Microbe Culture Medium Products Factory), beef extract (Sinopharm Chemical Reagent Co., Ltd), beef extract powder (MDBio, Inc), yeast extract powder (Beijing AoBoXing Bio-Tech Co., Ltd) at the above-mentioned culture conditions, and the β-caryophyllene products were detected.

#### Effect of concentration of organic nitrogen source

The shake-flask cultures were incubated in M9 minimal medium with different beef extract (solarbio) concentrations (1, 5, 10 or 15 g/L) at the above- mentioned culture conditions, and the β-caryophyllene products were detected.

#### Fed-batch fermentation

To further enhance the β-caryophyllene production from HAc, the fed-batch fermentation was carried out in a 5-L fermentor (BIOSTAT Bplus MO5L, Sartorius, Germany). To decrease the poisonousness of HAc on cells, a pH-coupled fed-batch fermentation was conducted as previously described by J.C. Liao [42]. The strain was grown in 2L fermentation medium. The temperature was controlled at 30 °C and the pH was maintained at ∼ 6.0 during the entire fermentation by adding pure HAc through an auto-pump. Cells were induced at an OD_600_ of ~ 4 using 0.25 mM IPTG. The residual HAc was measured via an enzyme kit (R-Biopharm). Then β-caryophyllene production was determined at a series of time points by GC as stated above. The biomass was determined by measuring the OD_600_ using a spectrophotometer (Cary 50 UV–Vis, Varian), where an OD_600_ of one corresponded to 0.43 g dry weight/L for *E. coli* BL21 strains.

Conversion efficiency (gram to gram) of HAc to β-caryophyllene was calculated with the following equation: Y = G_c_/G_h_ × 100 %.

Where Y = conversion efficiency (gram to gram, 100 %); G_c_ = weight of β-caryophyllene (g); G_h_ = weight of HAc (g).

## Abbreviations

DMAPP: dimethylallyl pyrophosphate
IPP: isopentenyl pyrophosphate
GPP: geranyl diphosphate
MVA: mevalonate
IPTG: isopropyl β-d-thiogalactoside
PCR: polymerase chain reaction
GC: gas chromatography
FPP: farnesyl diphosphate
